# The contribution of unpaid labour to poor mental health in the Swedish working population

**DOI:** 10.1016/j.ssmph.2025.101864

**Published:** 2025-09-18

**Authors:** Anu Molarius, Fredrik Granström, Jennifer Ervin

**Affiliations:** a Centre for Clinical Research, Region Värmland, Karlstad, Sweden; b Department of Public Health Sciences, Karlstad University, Karlstad, Sweden; c Department of Public Health, Region Värmland, Karlstad, Sweden; d Centre for Health Policy, Melbourne School of Population and Global Health, The University of Melbourne, Parkville, Victoria, Australia

**Keywords:** Unpaid labour, Mental health, Gender, Population studies, Sweden

## Abstract

Poor mental health is debilitating and of global public health concern. Unpaid labour has been recognised as an important yet neglected, and highly gendered, determinant of mental health. We examined the contribution of unpaid labour to poor mental health, in relation to other known risk factors, in employed adults. Study data is from 5462 employed Swedish women and men aged 30–49 years responding to a population survey in 2022. Independent associations between domestic work (inclusive of unpaid household and care work) and self-reported diagnosed depression and anxiety were examined by multiple logistic regression. The contributions of economic difficulties, loneliness, physical inactivity, risk consumption of alcohol and job dissatisfaction to depression and anxiety were also investigated. Gender-specific population attributable risks (PAR) were calculated to assess the contributions. In total, 10 % of the women and 6 % of the men reported depression while 12 % and 6 %, respectively, reported anxiety. Experiencing domestic work as burdensome explained 47 % (95 % CI: 31–57 %) (PAR) of the prevalence of depression in women, whereas no independent association was found in men. Loneliness, economic difficulties and physical inactivity accounted for 13–28 % of the prevalence of depression in both women and men. Burdensome domestic work was associated with anxiety in both genders and explained 22–25 % of the prevalence. Whilst longitudinal studies are needed, these results imply that unpaid labour is a highly important contributing factor to poor mental health, especially among women. Promoting a more equal division of unpaid labour has the potential to improve mental health across the working population.

## Highlights

• About half of the prevalence of diagnosed depression in women was attributable to burdensome domestic work.
• No independent association between burdensome domestic work and depression was found in men.
• Loneliness, economic difficulties and physical inactivity explained 13–28 % of the prevalence of depression.
• Burdensome domestic work explained 22–25 % of the prevalence of diagnosed anxiety for women and men.
• Unpaid labour is a highly important contributing factor to poor mental health in the working population.

## 1 Introduction

Mental health is a pressing public health concern worldwide, with depression and anxiety amongst the leading causes of disability globally (GBD 2019 Mental Disorders Collaborators, 2022; Moitra et al., 2023). Poor mental health is also the most common reason for sickness absence in the working population in Sweden (The Swedish Social Insurance Agency, 2024). Importantly, numerous social and economic determinants are known risk factors for mental health disorders. Many of them are potentially modifiable through policy and social reforms, meaning that targeted public policies have the power to effect health improvements (Alegria et al., 2018; Moitra et al., 2023). One such social determinant of mental health is unpaid labour (McMunn, 2023). Unpaid labour has been shown to be associated with greater mental health burden and negative effects on quality of life (Gilbert-Ouimet et al., 2020; Peristera et al., 2018; Seedat & Rondon, 2021). However, while factors such as financial hardship (Frankham et al., 2020) and loneliness (Mann et al., 2022) have been recognised as powerful risk factors for impaired mental health, unpaid labour has been historically under-researched.

Unpaid labour is typically conceptualised as all responsibilities and tasks done to maintain a household and/or it's family members without any monetary compensation. It includes all domestic work; household management and all types of unpaid caregiving (Ervin et al., 2022). Unpaid labour is highly gendered with women doing substantially more unpaid work than men despite the convergence of paid employment rates (OECD, 2018; Seedat & Rondon, 2021). It has been suggested that the higher risk of depressive and anxiety symptoms among women may be partially explained by this disproportionate burden (Seedat & Rondon, 2021). Indeed, a 2022 systematic review found high unpaid labour time to be associated with poorer mental health in employed women (Ervin et al., 2022). Differential exposure was considered a key reason as to why the effect was less apparent on men (Ervin et al., 2022). In addition to time spent on unpaid labour, Swedish research has also interrogated how the perceived burden of unpaid labour impacts mental health. It has shown that experiencing domestic work as burdensome is strongly associated with depression in both women and men (Molarius & Metsini, 2021, 2023).

Several theoretical frameworks have been proposed to explain the relationship between unpaid labour and health (Arber S et al., 1985; Nilsen et al., 2017; Tao et al., 2010). According to the *double burden theory*, combining paid work with high unpaid workloads is burdensome - also coined the ‘second shift’ by Hochschild in the late 20th century (Hochschild & Machung, 2012) - and leads to poorer health (Nilsen et al., 2017). A competing theory is the *expansion theory*, which posits that when women take on double roles in both the family and the labour market, the resulting broader social network, financial security and higher self-esteem can outweigh the strains of unpaid labour (Arber S et al., 1985). A limitation of both these theoretical frameworks is their gendered focus on women. A broader form of the double burden theory is *role strain theory* which implies that human energy is limited and that taking on increasing demands and/or multiple roles can have detrimental consequences for health (Hecht, 2001; Janzen & Kelly, 2012, pp. 67–82). Other related theories include the notion that unpaid labour consists of low-prestige activities that are not only physically demanding but mundane and isolating, leading to lower levels of mental wellbeing (Tao et al., 2010), as well as the *time poverty theory*, which refers to time scarcity arising from the disproportionate number of hours devoted each day to paid and unpaid work, leaving limited time for rest, leisure, or self-care (Artazcoz et al., 2024; Strazdins et al., 2011).

Nonetheless, despite recent attention to this historically neglected social determinant - partly due to the increased burden during the COVID pandemic - the impact of unpaid labour on health remains under-researched (McMunn, 2023; Seedat & Rondon, 2021). Moreover, given it is a ubiquitous and ongoing requirement of daily life for people everywhere, continuing to build the evidence base is vital, especially given the substantive population health implications and potential opportunity for mental health gains. From a public health perspective, it is important to gain insights into the contribution of social determinants on mental health at the population level. Notions of these contributions enable assessments of how much of the total burden of poor mental health (depression and anxiety) could be prevented if certain determinants could be eliminated. Such assessments can be done by calculating population-attributable risks (Greenland, 2015).

The main aim of this study was to examine the contribution of unpaid labour to poor mental health in employed adults and how the association between unpaid labour and mental health (self-reported diagnosed depression and anxiety) compares to other recognised determinants of mental health in the employed population; these being loneliness (Mann et al., 2022), economic difficulty (Frankham et al., 2020; Molarius & Granstrom, 2018), heavy alcohol consumption (Rehm et al., 2017), physical inactivity (Pearce et al., 2022), and job dissatisfaction (Faragher et al., 2005). We also investigated whether the associations between these exposures and mental health varied by gender.

## 2 Methods

### 2.1 Data source and sample

The study sample was derived from the 2022 Life & Health Survey; an extensive questionnaire sent to a random population sample in central Sweden in February–May 2022 (Region Värmland, 2022). Conducted approximately every five years, the aim of the survey is to monitor living conditions, lifestyle factors and health in the general population (Region Värmland, 2022). The sampling frame is the population register at Statistics Sweden, the statistical administrative authority in Sweden, covering all inhabitants of the study area. The area sampled covers five counties (Sörmland, Uppsala, Värmland, Västmanland and Örebro) including 55 municipalities with over one million inhabitants. In 2022, the questionnaire was sent to 78,117 persons aged 18 years and over. It was sent by mail and could be answered by mail or online. Data collection was discontinued after two postal reminders. Up to three short message service (SMS) reminders were also sent to persons with a registered mobile phone number. In total, 35,169 adults answered the questionnaire with an overall response rate of 45 %. The current study included a sub-sample of employed (including self-employed) women and men aged 30–49 years. This age-group was selected given it was considered the peak age bracket for women and men to be combining high amounts of unpaid work/care with paid employment. A total of 5462 subjects in this group answered the questionnaire including questions on unpaid labour and mental health.

### 2.2 Measures

#### 2.2.1 Outcomes

Two survey questions on mental health were used as outcomes. They were measured with the question “Do you have any of the following diagnosed illnesses?”. “Depression” was one of the two illnesses listed (with answer options no/yes) and was used to assess *self-reported diagnosed depression*. “Anxiety syndrome (e.g. panic disorder, generalized anxiety disorder, other anxiety disorder)” was the other of the two illnesses listed (with answer options no/yes) and was used to assess s*elf-reported diagnosed anxiety*.

#### 2.2.2 Exposures

##### 2.2.2.1 Unpaid labour

There were two questions pertaining to unpaid labour, here labelled domestic work (inclusive of unpaid household and care work). The first one was: “How many hours a week on average do you spend working at home that is not paid work? e.g. taking care of children, nursing relatives, buying groceries, cooking, paying the bills, washing the laundry, cleaning, taking care of a car, a house or a garden”. The categorical answer options were: 0–2, 3–10, 11–20, 21–30, and 31 or more hours per week. The measure was dichotomised into up to 20 h/week or greater than 20 h/week for the analyses. The cut-off value of 20 h/week aligns with previous studies (Krantz et al., 2005; Molarius & Metsini, 2021) and corresponds to a half-time working week.

The second question asked how often the respondent experienced domestic work as burdensome (never, seldom, sometimes, most of the time, all the time). These alternatives were condensed into two categories: “never/seldom”, and “sometimes/all or most of the time”.

##### 2.2.2.2 Social factors

Economic difficulties were estimated with the question “During the last 12 months, have you ever had difficulty in managing the regular expenses for food, rent, bills etc.?” with answer options “No”, “Yes, once”, “Yes, more than once”. The alternatives were dichotomised into no and yes.

Loneliness was measured with one question “Do you struggle with loneliness and isolation?”. The answer options were “No”, “Yes, mild problems” and “Yes, severe problems” and were dichotomised into no and yes.

##### 2.2.2.3 Lifestyle factors

Two questions, similar to those in the Swedish national public health survey (The Public Health Agency of Sweden, 2025) were used for measuring physical activity. These were 1) “How much time do you spend in a normal week on physical training that leaves you out of breath – for example running, fitness training, or ball sports?”, and 2) “How much time do you spend in a normal week on daily activities – for example walking, cycling, or gardening? Count all time together.”. Responses to these two questions were combined to determine whether the respondent reaches 150 activity minutes of moderate-to-vigorous physical activity per week as recommended by the WHO (World Health Organization, 2010), noting that the minutes spent in physical training (first question) were doubled when the sum of the minutes from the two questions was calculated in line with the definition in the Swedish national public health survey (The Public Health Agency of Sweden, 2025).

Alcohol consumption was measured using Alcohol Use Disorders Identification Test-C (AUDIT-C). It comprises three questions on the frequency and quantity of alcohol consumption. We used the following cut-offs for risk-drinking: 5 points for women and 6 points for men (The Public Health Agency of Sweden, 2025).

##### 2.2.2.4 Work factors

Job dissatisfaction was measured with the question “How satisfied are you with your current work?”. The answer categories were very satisfied, quite satisfied, neither satisfied nor unsatisfied, quite unsatisfied, very unsatisfied where the latter two options were categorised as dissatisfaction.

#### 2.2.3 Covariates

Educational level was obtained from a national education register and categorised into two levels: low (elementary school) or medium (upper secondary school), and high (at least 3 years of university or corresponding education). Family status was obtained from a survey question and categorised into living alone, living with partner, living with partner and children, and single parent.

#### 2.3 Statistical analyses

Differences in the distributions of background characteristics between women and men were tested using chi-square statistics. P-values <0.05 were considered as statistically significant. Multiple logistic regression analyses were conducted to assess variables independently associated with self-reported diagnosed depression and anxiety. The results from the logistic regression analyses are presented as adjusted odds ratios (OR) with 95 % confidence intervals (95 % CI). The multiple analyses were run separately by gender. All analyses were carried out using SPSS, version 28.0.

The concept of population-attributable risk (PAR) provides a valuable framework for assessing public health impact, as it estimates the proportion of disease burden (in our case, poor mental health) that could be prevented if specific social and economic issues could be eliminated. In our analysis, we calculated population attributable risks only for the variables that were found to be statistically significantly associated with depression or anxiety in the adjusted models. PARs were calculated using the following formula:

$$\text{PAR}=1-\left(\left(1-p_{c}\right)+\left(p_{c}/\text{OR}\right)\right)$$

where p_c_ is the proportion of exposed among cases of the outcome event and OR is the corresponding odds ratio from the adjusted logistic regression analysis (Greenland, 2015). 95 % confidence intervals for the PARs were calculated using the 95 % confidence interval estimates for the corresponding ORs.

## 3 Results

Table 1 presents the sample characteristics of the study population. More women than men had tertiary education. About 60 % of participants were living with a partner and children. The proportion of single parents was 10 % in women and 6 % in men. The prevalence of economic difficulties was about 13 % and loneliness 20 % for both men and women. The prevalence of spending more than 20 h per week on domestic work was 29 % in women and 20 % in men. A majority of both women (69 %) and men (54 %) experienced domestic work as burdensome at least sometimes. Slightly more men were physically inactive (31 %) than women (27 %). Risk drinking of alcohol was more common among men (15 %) than among women (9 %). Job dissatisfaction was reported by 7 % of participants. Less than 10 % reported diagnosed depression and about the same proportion reported diagnosed anxiety, but the prevalence of both diagnoses was higher among women than men.

**Table 1:** Sample characteristics employed participants 30–49 years.

		Total	Women	Men	p-value for difference between women and men
**N**		5 462	3 188	2 274	
	n	%	%	%	
**Covariates**					
**Educational level**					<0.001
Low/Medium	2 265	41.7	32.8	54.1	
High	3 173	58.3	67.2	45.9	
**Family status**					<0.001
Living with partner	1 008	18.5	18.8	17.9	
Living with partner and children	3 334	61.1	62.0	59.8	
Single parent	440	8.1	9.5	6.1	
Living alone	676	12.4	9.7	16.2	
**Risk factors**					
**Time spent in domestic work**					<0.001
0–20 h/w	4 064	74.5	70.6	80.0	
21+ h/w	1 388	25.5	29.4	20.0	
**Experiences domestic work as burdensome**					<0.001
Never/seldom	2 033	37.3	31.1	45.9	
Sometimes/all or most of the time	3 424	62.7	68.9	54.1	
**Economic difficulties during last 12 months**					0.02
No	722	86.8	85.9	88.0	
Yes	4 740	13.2	14.1	12.0	
**Loneliness**					0.91
No	4 374	80.1	80.1	80.2	
Yes	1 085	19.9	19.9	19.8	
**Physical inactivity**					<0.001
No	3 893	71.3	73.1	68.8	
Yes	1 566	28.7	26.9	31.2	
**Risk drinking of alcohol**					<0.001
No	4 771	88.7	91.3	85.0	
Yes	609	11.3	8.7	15.0	
**Job dissatisfaction**					
No	5 059	93.1	93.0	93.3	0.71
Yes	374	6.9	7.0	6.7	
**Outcomes**					
**Diagnosed depression**					<0.001
Yes	439	8.0	9.5	6.0	
No	5 015	92.0	90.5	94.0	
**Diagnosed anxiety**					<0.001
Yes	516	9.5	11.8	6.1	
No	4 941	90.5	88.2	93.9	

Table 2 gives the crude and adjusted ORs related to depression. After adjusting for educational level, family status and the other major risk factors, economic difficulties, loneliness, and physical inactivity were independently associated with self-reported depression in both women and men (Table 2). Experiencing domestic work as burdensome and job dissatisfaction were independently associated with depression in women only. Domestic work time and risk drinking of alcohol were not independently associated with depression in either gender.

**Table 2:** Crude and adjusted odds ratios (95 % confidence intervals in parentheses) for self-reported diagnosed depression, employed participants 30–49 years.

	Women		Men	
	Crude OR	Adjusted OR^a^	Crude OR	Adjusted OR^a^
**Time spent in domestic work**				
0–20 h/w	1	1	1	1
21+ h/w	1.0 (0.8, 1.3)	0.9 (0.7, 1.3)	0.8 (0.5, 1.2)	0.9 (0.6, 1.5)
**Experiences domestic work as burdensome**				
Never/seldom	1	1	1	1
Sometimes/all or most of the time	**2.4 (1.7, 3.2)**	**2.2 (1.6, 3.1)**	**1.6 (1.1, 2.3)**	1.4 (0.9, 2.0)
**Economic difficulties during last 12 months**				
No	1	1	1	1
Yes	**3.4 (2.6, 4.4)**	**2.2 (1.7, 3.0)**	**2.8 (1.8, 4.2)**	**2.1 (1.3, 3.3)**
**Loneliness**				
No	1	1	1	1
Yes	**3.9 (3.0, 5.0)**	**2.7 (2.1, 3.6)**	**2.9 (2.0, 4.2)**	**1.8 (1.2, 2.8)**
**Physical inactivity**				
No	1	1	1	1
Yes	**1.9 (1.5, 2.5)**	**1.5 (1.2, 2.0)**	**1.7 (1.2, 2.5)**	**1.5 (1.0, 2.2)**
**Risk drinking of alcohol**				
No	1	1	1	1
Yes	1.1 (0.8, 1.7)	1.0 (0.6, 1.5)	1.5 (1.0, 2.3)	1.4 (0.9, 2.2)
**Job dissatisfaction**				
No	1	1		1
Yes	**2.1 (1.5, 3.1)**	**1.6 (1.1, 2.4)**	**2.4 (1.5, 4.1)**	1.6 (0.9, 2.8)

Statistically significant ORs are shown in bold type.

a Adjusted for all given variables and for educational level and family status.

After adjustment for covariates, experiencing domestic work as burdensome, loneliness, and job dissatisfaction were independently associated with self-reported anxiety in both women and men (Table 3). Economic difficulties and physical inactivity were associated with anxiety only among women. Domestic work time and risk drinking of alcohol were not independently associated with anxiety in either gender.

**Table 3:** Crude and adjusted odds ratios (95 % confidence intervals in parentheses) for self-reported diagnosed anxiety, employed participants 30–49 years.

	Women		Men	
	Crude OR	Adjusted OR^a^	Crude OR	Adjusted OR^a^
**Time spent in domestic work**				
0–20 h/w	1	1	1	1
21+ h/w	1.2 (1.0, 1.5)	1.1 (0.9, 1.5)	0.8 (0.5, 1.2)	0.9 (0.6, 1.5)
**Experiences domestic work as burdensome**				
Never/seldom	1	1		1
Sometimes/all or most of the time	**1.6 (1.2, 2.1)**	**1.4 (1.1, 1.8)**	**1.8 (1.2, 2.5)**	**1.5 (1.0, 2.3)**
**Economic difficulties during last 12 months**				
No	1	1	1	1
Yes	**3.0 (2.3, 3.8)**	**2.2 (1.7, 2.9)**	**1.9 (1.2, 2.9)**	1.3 (0.8, 2.1)
**Loneliness**				
No	1	1		1
Yes	**2.7 (2.1, 3.4)**	**2.0 (1.6, 2.6)**	**2.9 (2.0, 4.1)**	**2.0 (1.3, 3.0)**
**Physical inactivity**				
No	1	1		1
Yes	**1.6 (1.3, 2.1)**	**1.4 (1.1, 1.7)**	**1.6 (1.1, 2.3)**	1.4 (1.0, 2.1)
**Risk drinking of alcohol**				
No	1	1	1	1
Yes	1.0 (0.7, 1.5)	0.9 (0.6, 1.4)	1.4 (0.9, 2.2)	1.4 (0.9, 2.2)
**Job dissatisfaction**				
No	1	1		1
Yes	**2.0 (1.4, 2.9)**	**1.6 (1.1, 2.4)**	**2.9 (1.8, 4.7)**	**2.0 (1.2, 3.4)**

Statistically significant ORs are shown in bold type.

a Adjusted for all given variables and for educational level and family status.

Table 4 presents the population attributable risks for self-reported diagnosed depression and anxiety in working women and men aged 30–49 years. Experiencing domestic work as burdensome (sometimes, most or all of the time) explained 47 % (95 % CI: 31–57 %) of the prevalence of self-reported diagnosed depression in employed women. Since it was not independently associated with depression in employed men the corresponding proportion was not calculated for men. Loneliness explained 28 % (95 % CI: 24–32 %) of the prevalence of depression in women and 18 % (95 % CI: 7–25 %) in men, whereas economic difficulties accounted for 17 % (95 % CI: 13–21 %) and 13 % (95 % CI: 6–18 %), for women and men respectively. 13 % (95 % CI: 7–20 %) in women and 14 % (95 % CI: 4–24 %) in men was attributable to physical inactivity. Job dissatisfaction accounted for 5 % (95 % CI: 1–7 %) in women.

**Table 4:** Population attributable risks (PAR) for self-reported diagnosed depression and anxiety, employed participants30-49 years.

	Women			Men		
	p_c_^a^	OR^b^ (95 % CI)	PAR (95 % CI) %	p_c_^a^	OR^b^ (95 % CI)	PAR (95 % CI) %
**Diagnosed depression**						
Experiences domestic work as burdensome	0.831	2.3 (1.6, 3.2)	47 (31, 57)	–	–	–
Economic difficulties during last 12 months	0.318	2.2 (1.7, 3.0)	17 (13, 21)	0.257	2.1 (1.3, 3.3)	13 (6, 18)
Loneliness	0.449	2.7 (2.1, 3.5)	28 (24, 32)	0.400	1.8 (1.2, 2.7)	18 (7, 25)
Physical inactivity	0.397	1.5 (1.2, 2.0)	13 (7, 20)	0.434	1.5 (1.1, 2.2)	14 (4, 24)
Job dissatisfaction	0.126	1.6 (1.1, 2.3)	5 (1, 7)	–	–	–

**Diagnosed anxiety**						
Experiences domestic work as burdensome	0.771	1.4 (1.1, 1.9)	22 (7, 37)	0.669	1.6 (1.1, 2.4)	25 (6, 39)
Economic difficulties during last 12 months	0.291	2.2 (1.7, 2.9)	16 (12, 19)	–	–	–
Loneliness	0.364	2.0 (1.6, 2.6)	18 (14, 22)	0.394	1.9 (1.3, 2.8)	19 (9, 25)
Physical inactivity	0.362	1.4 (1.1, 1.7)	10 (3, 15)	–	–	–
Job dissatisfaction	0.120	1.7 (1.1, 2.4)	5 (1, 7)	0.159	2.0 (1.2, 3.3)	8 (3, 11)

- Not statistically significant in multiple logistic regression analysis.

a Proportion exposed among cases.

b Adjusted odds ratio from logistic regression analysis including educational level, family status, economic difficulties, loneliness, burdensome domestic work, physical inactivity and job dissatisfaction.

Experiencing domestic work as burdensome explained 22 % (95 % CI: 7–37 %) of the prevalence of self-reported diagnosed anxiety in working women and 25 % (95 % CI: 6–39 %) in working men (Table 4). Loneliness and, among women, economic difficulties and physical inactivity accounted for 10–19 % each. The contribution of job dissatisfaction was 5–8 %.

## 4 Discussion

Using Swedish data, we examined the contribution of unpaid labour to poor mental health amongst working adults (aged 30–49 years) at the population level in relation to other known risk factors. Our findings showed that experiencing domestic work as burdensome explained approximately half of the prevalence of self-reported diagnosed depression in women, whereas no independent association was found in men. The proportions explained by loneliness, economic difficulties and physical inactivity were smaller but statistically significant in both women and men. Burdensome domestic work was also associated with self-reported diagnosed anxiety in both genders and accounted for about a quarter of the prevalence of anxiety. Regarding key gender differences, we found that women spent more time in domestic work and more often experienced it as burdensome than men did. In addition, the prevalence of both depression and anxiety was higher among women than among men.

To our knowledge, this study is the first to estimate the PAR of unpaid labour on mental health disorders in the working population. Previous studies have used PARs (or population attributable fractions PAFs) to investigate, for example, the contribution of poverty to common mental disorders in the UK (Thomson et al., 2023), and the effect of economic crisis on mental health in Spain (Gili et al., 2013). Furthermore, a recent meta-umbrella systematic review quantified the global PAF of potentially modifiable risk factors for mental disorders and, whilst no included studies examined unpaid labour, substantive research investigated paid work (Dragioti et al., 2022). Indeed, one of the largest PAFs was for a paid work risk factor - job strain and depression (18 %) – emerging as a core modifiable target among working adults (Dragioti et al., 2022). Parallels can be drawn between job strain (high job demands, low job control) and unpaid labour. Both are thought to impart mental health implications through stress process model pathways (Brunner, 1997; Pearlin et al., 1981; Schneiderman et al., 2005), with role strain and time scarcity mechanisms thought to be key contributors to chronic stress in working populations doing high amounts of unpaid labour (Artazcoz et al., 2024; Hecht, 2001; Janzen & Kelly, 2012, pp. 67–82; Strazdins et al., 2016). Accordingly, our findings - that poor mental health among working adults is highly attributable to experiencing unpaid labour as burdensome - are consistent with both role strain and time poverty theories.

With respect to mental health among employed adults more generally, the findings of a 2022 systematic review of the literature indicated that unpaid labour time is negatively associated with women's mental health, with effects less apparent for men (Ervin et al., 2022). Its findings contrast with our Swedish working population results where spending more than 20 h/week in unpaid work was not associated with depression. Given the cross-sectional nature of our study, reverse causation may have contributed to our null finding, or it may be that our measure of total domestic work (household work and care) hides the nuance between different types of unpaid labour. For example, a 2023 longitudinal Australian study reported no association between total unpaid labour time and mental health, and this was attributed to opposing effect directions between contributing domains of unpaid labour (Ervin et al., 2023). Their finding that hours spent in housework was negatively associated with mental health, but the opposite was true for other domains such outdoor tasks (Ervin et al., 2023), aligns with the notion that different types of unpaid labour may differ in degree of strain and control over tasks and thus have different implications for mental health. Low-schedule-control tasks, such as laundry and cooking, must be performed daily and at certain times, whereas high-schedule-control tasks, such as outdoor tasks and car maintenance can often be done without any time urgency (Tao et al., 2010). Women often spend more hours in low-schedule-control tasks whereas men spend more hours in high-schedule-control tasks. In addition, the division of unpaid labour in the household can also contribute to the degree of strain of the tasks experienced. For example, longitudinal research in South Korea found that a 1 h increase in husbands' housework was linked to a 0.88-fold decrease in the risk of depressive symptom onset in women (Baek et al., 2024).

In comparing unpaid labour to other recognised determinants of mental health in the employed population, our study confirmed the strong association between financial strain/difficulties and poor mental health (Frankham et al., 2020), and the known associations between loneliness (Mann et al., 2022), physical activity (Pearce et al., 2022) and job dissatisfaction (Faragher et al., 2005) and mental health outcomes. Importantly, however, we found the contribution of these factors to the prevalence of depression among employed women to be smaller than the contribution of burdensome domestic work. Contradicting previous literature (Rehm et al., 2017), we did not find any association between risk drinking of alcohol and depression or anxiety.

The results from the PAR analyses suggest that burdensome domestic work is a highly contributing independent factor of depression in employed prime working-age women and a contributing factor for anxiety in both genders. Interestingly, when we changed the cut-off for burdensome domestic work to only include those experiencing domestic work as burdensome most or all the time, it was independently associated with depression also among men (adjusted OR 2.6 95 % CI: 1.6, 4.1) and explained 12 % of depression among men. This suggests that burdensome domestic work also contributes to depression in employed men, but the contribution is smaller than in women and restricted to a subgroup who most often experience domestic work as burdensome.

It is notable that the comparatively high contribution (PAR) of burdensome domestic work to poor mental health found in this study is largely attributable to the high prevalence of unpaid labour in this working population. Whilst it is not feasible to fully erase domestic requirements from daily life, more effort is needed to reduce the burden of unpaid domestic work and drive greater equity in the division of labour. Despite Sweden's progressive social policies (e.g. generous gender-neutral parental leave and reduced working hours options) facilitating a more equal division of paid and unpaid work than in many other countries, uptake of these opportunities remains imbalanced. Striving towards a dual-earner/dual-caregiver society in which men and women participate symmetrically in paid work and unpaid labour/caregiving would deliver many benefits (Gornick & Meyers, 2008), and has the potential to improve the mental health of the working population, especially among women.

The individual and societal burden of poor mental health – including the costs for health care, medication and sickness absence – is high (Ekman et al., 2013). Moreover, it is higher among women than among men (Molarius & Metsini, 2021). Yet, unpaid labour, despite being a daily reality worldwide, remains a neglected determinant of mental health (McMunn, 2023). Our findings, together with previous literature, highlight unpaid labour as a substantial contributor to poor mental health in the working population, especially among women. This underscores the need to design social policies to reduce the burden of unpaid domestic work and to strive for a more equal division of paid and unpaid work between women and men. This is especially pertinent given that the gender gap in unpaid labour is even wider in many countries compared with Sweden (OECD Family Database, 2016). Ultimately, there is a need to make unpaid labour and its association with mental health more visible to public health scientists and policymakers.

## 5 Strengths and limitations

The current study has several strengths, being population-based and including a large sample (N = 5462) of employed women and men of prime working age. Several known risk factors for poor mental health could be compared. Two different outcomes were used to measure mental health, and these were for diagnosed depression and anxiety rather than just mental health symptomology. Population attributable risks (fractions) were used to measure the contribution of unpaid labour to depression and anxiety in employed persons. PARs consider both the strength of the association between the risk factor and the outcome and the prevalence of the risk factor - and express the fraction of the outcome in the target population that could be prevented if the risk factor was removed. Miettinen's formula, further developed by Greenland (Greenland, 2015), was used to investigate unbiased PARs using adjusted ORs.

The main limitation of this study is the cross-sectional design, which prevents any interpretations about causality. It is probable that the associations of interest are bi-directional i.e. that unpaid work increases the risk of depression and anxiety, but also that depression and anxiety increase the risk of experiencing domestic work as burdensome. Importantly, the use of PAR presupposes a causal association between the exposure and the outcome and that the exposure is amenable to intervention (Rockhill et al., 1998). As such, the quantitative results of the current study should be interpreted with caution. Nonetheless, the few longitudinal studies on this association have found a longitudinal effect of high unpaid workload on mental health in the working-age population (Ervin et al., 2023; Gilbert-Ouimet et al., 2020; Peristera et al., 2018).

Secondly, both our outcome variables and information on unpaid work were self-reported and therefore susceptible to common method bias. Our mental health outcome variables - depression and anxiety - refer to diagnosed illnesses. Whilst previous studies have found that self-reported clinician diagnosed depression is of adequate validity to measure depression (Maske et al., 2017; Sanchez-Villegas et al., 2008), the validity of the diagnosed anxiety measure used in this study has not been investigated. Another limitation regards the dichotomisation of the independent variables which might reduce the specificity of the data. However, a previous study examining the association between burdensome domestic work and depression did demonstrate a dose-response relationship (Molarius & Metsini, 2023).

A further limitation relates to the validity of the measures of domestic work. Notably, whilst the other included determinants (loneliness, economic difficulties, physical inactivity and job dissatisfaction) are well-established risk factors for poor mental health and can be assessed using validated measures, unpaid labour lacks both a strong theoretical foundation and robust measurement tools, reflecting the limited scientific attention it has received. This methodological underdevelopment underscores the importance of standardising the way in which different dimensions and types of unpaid labour are measured. However, the strong association we found between burdensome domestic work and diagnosed depression in women is in line with other studies showing strong negative associations between strain/demands in domestic work and mental health (Maeda et al., 2019; Staland-Nyman et al., 2008).

Furthermore, we concede that some factors which may influence the associations observed (such as the type of unpaid work, number of children, or work stress) were not measured in the current study. Additionally, we were not able to control for employment level (e.g. working part-time or full-time). Given that some of our sample likely work part-time hours, it is feasible that the associations we found might be stronger if we were able to restrict our sample to those in full time employment, noting that in 2022, 20 % of employed women and 8 % of employed men aged 35–44 years worked part time in Sweden (Statistics Sweden, 2024). Lastly, whilst the overall response rate of the study was 45 %, the response rate specifically among employed persons 30–49 years is not known.

## 6 Conclusion

Whilst more research in this area is required, including robust longitudinal studies and validated tools for assessing unpaid labour, our results suggest that if the burden of unpaid labour on women was relieved, the prevalence of depression in working women could be substantially reduced. In addition, a considerable proportion of anxiety in both working women and men might be avoided. Addressing the unequal division of unpaid labour - particularly the disproportionate burden borne by women - is a key policy priority, with the potential to advance gender equality and yield substantial improvements in the mental health of the working population.

## CRediT authorship contribution statement

**Anu Molarius:** Writing – review & editing, Writing – original draft, Validation, Methodology, Formal analysis, Conceptualization. **Fredrik Granström:** Writing – review & editing, Validation, Methodology. **Jennifer Ervin:** Writing – review & editing, Writing – original draft, Conceptualization.

## Ethical statement

The data on gender, age, and educational level are based on registry data from Statistics Sweden. By answering the questionnaire, participants gave their informed consent that questionnaire data would be linked to Swedish official registers at Statistics Sweden through personal identification numbers. After the record linkage, all identity information was removed before the material was handed over from Statistics Sweden to the regions for further processing. Authorization from the Swedish Ethical Review Authority has been obtained (Dnr 2021-05814-01). The data material is protected according to the Public and Privacy Act (2009: 400, Chapter 24, Section 8).

The data materials generated and/or analysed during the current study are not publicly available due to confidentiality and regulations under the Swedish law (Public and Privacy Act 2009:400, Chapter 24, Section 8), but descriptive data in table form are available from the corresponding author on reasonable request.

## Declaration of interest statement

We declare no competing interests.

## Data Availability

The data that has been used is confidential.

## References

[bib1] Alegria M., NeMoyer A., Falgas Bague I., Wang Y., Alvarez K. (2018). Social determinants of mental health: Where we are and where we need to Go. Current Psychiatry Reports.

[bib2] Arber S., Gilbert G.N., Dale A. (1985). Paid employment and women's health: A benefit or a source of role strain?. Sociology of Health & Illness.

[bib3] Artazcoz L., Cortes-Franch I., Arcas M.M., Olle-Espluga L., Perez K. (2024). Time poverty, health and health-related behaviours in a southern European city: A gender issue. Journal of Epidemiology & Community Health.

[bib4] Baek S.U., Lee Y.M., Won J.U., Yoon J.H. (2024). Association between husband's participation in household work and the onset of depressive symptoms in married women: A population-based longitudinal study in South Korea. Social Science & Medicine.

[bib5] Brunner E. (1997). Socioeconomic determinants of health: Stress and the biology of inequality. Brittish Medical Journal.

[bib6] Dragioti E., Radua J., Solmi M., Arango C., Oliver D., Cortese S. (2022). Global population attributable fraction of potentially modifiable risk factors for mental disorders: A meta-umbrella systematic review. Molecular Psychiatry.

[bib7] Ekman M., Granström O., Omérov S., Jacob J., Landén M. (2013). The societal cost of depression: Evidence from 10,000 Swedish patients in psychiatric care. Journal of Affective Disorders.

[bib8] Ervin J., Taouk Y., Fleitas Alfonzo L., Hewitt B., King T. (2022). Gender differences in the association between unpaid labour and mental health in employed adults: A systematic review. The Lancet Public Health.

[bib9] Ervin J., Taouk Y., Hewitt B., King T. (2023). The association between unpaid labour and mental health in working-age adults in Australia from 2002 to 2020: A longitudinal population-based cohort study. The Lancet Public Health.

[bib10] Faragher E.B., Cass M., Cooper C.L. (2005). The relationship between job satisfaction and health: A meta-analysis. Occupational and Environmental Medicine.

[bib11] Frankham C., Richardson T., Maguire N. (2020). Psychological factors associated with financial hardship and mental health: A systematic review. Clinical Psychology Review.

[bib12] GBD 2019 Mental Disorders Collaborators (2022). Global, regional, and national burden of 12 mental disorders in 204 countries and territories, 1990–2019: A systematic analysis for the global burden of Disease study 2019. The Lancet Psychiatry.

[bib13] Gilbert-Ouimet M., Brisson C., Vezina M. (2020). Psychosocial work stressors, high family responsibilities, and psychological distress among women: A 5-year prospective study. American Journal of Industrial Medicine.

[bib14] Gili M., Roca M., Basu S., McKee M., Stuckler D. (2013). The mental health risks of economic crisis in Spain: Evidence from primary care centres, 2006 and 2010. The European Journal of Public Health.

[bib15] Gornick J.C., Meyers M.K. (2008). Creating gender egalitarian societies: An agenda for reform. Politics & Society.

[bib16] Greenland S. (2015). Concepts and pitfalls in measuring and interpreting attributable fractions, prevented fractions, and causation probabilities. Annals of Epidemiology.

[bib17] Hecht L.M. (2001). Role conflict and role overload: Different concepts, different consequences. Sociological Inquiry.

[bib18] Hochschild A., Machung A. (2012).

[bib19] Janzen B., Kelly I.W. (2012). Psychological distress: Symptoms, causes and coping.

[bib20] Krantz G., Berntsson L., Lundberg U. (2005). Total workload, work stress and perceived symptoms in Swedish male and female white-collar employees. The European Journal of Public Health.

[bib21] Maeda E., Nomura K., Hiraike O., Sugimori H., Kinoshita A., Osuga Y. (2019). Domestic work stress and self-rated psychological health among women: A cross-sectional study in Japan. Environmental Health and Preventive Medicine.

[bib22] Mann F., Wang J., Pearce E., Ma R., Schlief M., Lloyd-Evans B. (2022). Loneliness and the onset of new mental health problems in the general population. Social Psychiatry and Psychiatric Epidemiology.

[bib23] Maske U.E., Hapke U., Riedel-Heller S.G., Busch M.A., Kessler R.C. (2017). Respondents' report of a clinician-diagnosed depression in health surveys: Comparison with DSM-IV mental disorders in the general adult population in Germany. BMC Psychiatry.

[bib24] McMunn A. (2023). Unpaid labour is a neglected social determinant of health. The Lancet Public Health.

[bib25] Moitra M., Owens S., Hailemariam M., Wilson K.S., Mensa-Kwao A., Gonese G. (2023). Global mental health: Where we are and where we are going. Current Psychiatry Reports.

[bib26] Molarius A., Granstrom F. (2018). Educational differences in psychological distress? Results from a population-based sample of men and women in Sweden in 2012. BMJ Open.

[bib27] Molarius A., Metsini A. (2021). Domestic work, self-reported diagnosed depression and related costs among women and men-results from a population-based study in Sweden. International Journal of Environmental Research and Public Health.

[bib28] Molarius A., Metsini A. (2023). The Association between time spent in domestic work and mental health among women and men. International Journal of Environmental Research and Public Health.

[bib29] Nilsen W., Skipstein A., Østby K.A., Mykletun A. (2017). Examination of the double burden hypothesis-a systematic review of work-family conflict and sickness absence. The European Journal of Public Health.

[bib30] OECD (2018).

[bib31] OECD Family Database (2016). Time used for work, care and daily household chores. LMF2.5. https://webfs.oecd.org/Els-com/Family_Database/LMF2_5_Time_use_of_work_and_care.pdf.

[bib32] Pearce M., Garcia L., Abbas A., Strain T., Schuch F.B., Golubic R. (2022). Association between physical activity and risk of depression: A systematic review and meta-analysis. JAMA Psychiatry.

[bib33] Pearlin L.I., Menaghan E.G., Lieberman M.A., Mullan J.T. (1981). The stress process. Journal of Health and Social Behavior.

[bib34] Peristera P., Westerlund H., Magnusson Hanson L.L. (2018). Paid and unpaid working hours among Swedish men and women in relation to depressive symptom trajectories: Results from four waves of the Swedish Longitudinal Occupational Survey of Health. BMJ Open.

[bib35] Region Värmland (2022). Liv & hälsa 2022 - En undersökning om livsvillkor, levnadsvanor och hälsa. [Life & health 2022 – A survey of living conditions, lifestyle habits and health].

[bib36] Rehm J., Gmel G.E.S., Gmel G., Hasan O.S.M., Imtiaz S., Popova S. (2017). The relationship between different dimensions of alcohol use and the burden of disease-an update. Addiction.

[bib37] Rockhill B., Newman B., Weinberg C. (1998). Use and misuse of population attributable fractions. American Journal of Public Health.

[bib38] Sanchez-Villegas A., Schlatter J., Ortuno F., Lahortiga F., Pla J., Benito S. (2008). Validity of a self-reported diagnosis of depression among participants in a cohort study using the structured clinical interview for DSM-IV (SCID-I). BMC Psychiatry.

[bib39] Schneiderman N., Ironson G., Siegel S.D. (2005). Stress and health: Psychological, behavioral, and biological determinants. Annual Review of Clinical Psychology.

[bib40] Seedat S., Rondon M. (2021). Women's wellbeing and the burden of unpaid work. Brittish Medical Journal.

[bib41] Staland-Nyman C., Hensing G., Alexanderson K. (2008). Associations between strain in domestic work and self-rated health: A study of employed women in Sweden. Scandinavian Journal of Public Health.

[bib42] Statistics Sweden (2024). Employed by age and agreed working hours as full-time and part-time. Years 2021 - 2024. https://www.statistikdatabasen.scb.se/pxweb/sv/ssd/START__LE__LE0201__LE0201EKO/Tema225N/table/tableViewLayout1/.

[bib43] Strazdins L., Griffin A.L., Broom D.H., Banwell C., Korda R., Dixon J. (2011). Time scarcity: Another health inequality?. Environment and Planning A.

[bib44] Strazdins L., Welsh J., Korda R., Broom D., Paolucci F. (2016). Not all hours are equal: Could time be a social determinant of health?. Sociology of Health & Illness.

[bib45] Tao W., Janzen B.L., Abonyi S. (2010). Gender, division of unpaid family work and psychological distress in dual-earner families. Clinical Practice and Epidemiology in Mental Health.

[bib46] The Public Health Agency of Sweden (2025). https://www.folkhalsomyndigheten.se/.

[bib47] The Swedish Social Insurance Agency (2024). Psykisk ohälsa i dagens arbetsliv. Försäkringskassans lägesrapport.

[bib48] Thomson R.M., Kopasker D., Leyland A., Pearce A., Katikireddi S.V. (2023). Effects of poverty on mental health in the UK working-age population: Causal analyses of the UK household longitudinal study. International Journal of Epidemiology.

[bib49] World Health Organization (2010).

